# Orthographic representations in American Sign Language fingerspelling

**DOI:** 10.1017/s0142716426100617

**Published:** 2026-05-08

**Authors:** Zed Sehyr, Lauren Fillet, Kiana Billot-Vasquez, Emily Saunders, Karen Emmorey

**Affiliations:** 1 Department of Communication Sciences and Disorders, Chapman University, USA; 2 School of Education and Information Studies, University of California, Los Angeles, USA; 3 Wheelock College of Education and Human Development, Boston University, USA; 4 School of Speech, Language, and Hearing Sciences, San Diego State University, USA

**Keywords:** American Sign Language, Orthography, Fingerspelling, Writing, Deaf

## Abstract

This study examined fingerspelling accuracy and error patterns in deaf American Sign Language (ASL) signers and written spelling in hearing English speakers to investigate how orthographic representations are shaped by phonological, visual, and motor encoding strategies. Deaf participants (*n* = 39) completed a fingerspelling repetition task, while hearing participants (*n* = 35) completed a written dictation task using the same word and pseudoword stimuli. While overall accuracy did not differ significantly between groups, deaf participants exhibited qualitatively distinct error patterns, including higher rates of deletions and transpositions, compared to hearing participants who made more substitution errors. Deaf participants also produced more pronunciation-violating errors. Notably, they showed greater accuracy in preserving geminate (double letter) segments, highlighting enhanced sensitivity to letter identity and quantity, likely supported by the explicit visual-motor representation of geminates in fingerspelling. Additionally, deaf participants showed no difference in accuracy between real words and pseudowords, indicating that fingerspelling strategies generalized beyond stored lexical forms. We interpret these findings in light of graphemic buffer constraints, motoric fluency pressures, and the structural affordances of the fingerspelling system. Visually based encoding strategies can support robust orthographic representations for those who rely less on speech-based phonological coding.

American Sign Language (ASL) fingerspelling represents a dynamic visual–manual system in which each letter of an English word is encoded as a distinct handshape configuration. But far from being a mere representation of print, fingerspelling is an integral part of the ASL lexicon and plays a central role in bridging signed and written modalities (Haptonstall-Nykaza & Schick, 2007; Padden & Le Master, 1985; Stone et al., 2015). Because it maps orthographic forms onto visual-motor sequences without reliance on speech-based phonology, fingerspelling offers a valuable opportunity to investigate how orthographic representations, such as letter identity, position, and quantity, are encoded, maintained, and reproduced across modalities.

Patterns of spelling errors are an important diagnostic of orthographic precision and retrieval (Andrews & Hersch, 2010; Buchwald & Rapp, 2004). Unlike hearing speakers, whose spelling strategies are typically rooted in speech-based phonology, deaf ASL signers are exposed to fingerspelling in natural discourse well before mastering written spelling (Padden, 2006). Fingerspelling may provide an alternative route to orthographic knowledge, shaped by visual or articulatory rather than auditory phonological experience (Brentari & Padden, 2001; Lee & Secora, 2022; Padden, 2006). Prior research on spelling in deaf writers has highlighted differences in orthographic representations (Gärdenfors et al., 2019; Hanson et al., 1983; Olson & Caramazza, 2004) but has rarely addressed how these representations manifest in fingerspelling production. Moreover, past accounts have too often adopted a deficit perspective, attributing errors primarily to reduced phonological access or poor phonological skill rather than considering how alternative encoding strategies might support orthographic skill.

Despite its theoretical and practical importance, systematic investigations of fingerspelling error patterns remain scarce. In this study, we examine error types and frequency in a fingerspelling reproduction task completed by deaf ASL signers, using a classification system adapted from Olson and Caramazza (2004), which includes deletions, insertions, substitutions, transpositions, doubling (geminate) errors, and speech-based phonological violations. By analyzing these patterns, we explore how orthographic sequences are produced under visual–manual encoding conditions, with implications for working memory mechanisms (e.g., a “graphemic buffer”).

## Background

Recent studies of written spelling have shown that deaf signers can achieve accuracy comparable to hearing peers, especially if they have early access to sign language (Daigle et al., 2020; Gärdenfors et al., 2019; Miller et al., 2021; Vizzi et al., 2022). These findings challenge deficit-based views that attribute lower spelling performance in deaf individuals to incomplete access to speech-based phonology. However, deaf and hearing participants with matched reading levels differ qualitatively on error types (Daigle et al., 2020), suggesting that alternative strategies, potentially those grounded in visual or manual processing, may support orthographic development in deaf signers. For example, a visual similarity bias can drive transpositions or deletions of similar-looking letters (e.g., “dook” for “book” or “worb” for “word”) or letters from visually similar segments (e.g., deleting “a” from the “ea” segment in “disappearance”), see Table 1 for error types and examples. On the other hand, visually motivated strategies lead to selective preservation of visually salient silent letters in deaf participants but greater deletion in hearing participants (e.g., the “w” in “snow” Aaron et al., 1998). This tendency may extend to fingerspelling, where similar-looking or manually similar handshapes could contribute to confusion. Gärdenfors et al. (2019) reported that Swedish deaf signers wrote “sengen” for “sängen” [“the bed”], reflecting a substitution likely influenced by visual similarity in fingerspelled handshapes for “ä” and “e” in both print and fingerspelling in Swedish Sign Language. Gärdenfors et al. (2019) suggest that fingerspelling enables deaf learners to adopt strategies such as “spell as it looks” which promotes the development of strong visual-orthographic representations.

Fingerspelling may reinforce visually based orthographic strategies by explicitly representing letter sequences through movements and handshapes. However, use of fingerspelling may also reduce the salience of precise letter positions due to transitional movements and coarticulation present in dynamic fingerspelling (Keane & Brentari, 2016). This combination of visual strength and greater flexibility on positional letter coding may explain why certain types of errors, such as letter transpositions and letter shifts, are more prevalent in deaf signers’ spelling (Olson & Caramazza, 2004). Whether such error types also emerge in fingerspelling productions remains an open question.

Written spelling studies have shown that deaf signers often produce errors that violate English phonology while maintaining orthographic permissibility. For example, Olson and Caramazza (2004) found that 80% of errors made by deaf participants violated pronunciation, compared to only 27% of errors by hearing participants. These included deletions (e.g., “rass” for “brass”) and transpositions (e.g., “secert” for “secret”), reflecting reduced reliance on phoneme-to-grapheme mappings and increased sensitivity to visual orthography (Aaron et al., 1998; Hanson, 1982; Olson & Caramazza, 2004). Deaf participants also tend to replace unfamiliar or irregular letter sequences with more common ones, suggesting influence from visual familiarity rather than phonological structure.

Phonological information is not entirely absent from deaf signers’ spelling representations; both deaf and hearing participants show shared sensitivity to phonological structure and complexity. For example, both groups make fewer errors on simple than complex syllables and tend to delete interior consonants (those closest to the vowel) in unstressed syllables (Olson and Caramazza, 2004). These patterns suggest that reduced phonological access alone may not fully account for deaf signers’ distinct spelling error patterns. In addition, deaf signers utilize speech-based phonology in short-term memory tasks when recalling fingerspelled word lists (Sehyr et al., 2017), suggesting a link between fingerspelled and phonological representations. Supporting this link, English mouthing often accompanies fingerspelling in natural signing contexts (Nadolske & Rosenstock, 2007) and may provide visual phonological cues to orthographic structure (Emmorey & Petrich, 2012). Nevertheless, the use of speech-based phonological strategies in written and/or fingerspelled production in deaf signers may be limited and variable across signers. It remains to be seen to what extent speech-based phonological strategies influence fingerspelling error patterns.

Geminates (e.g., double letters; “bb” in “rabbit”) offer a great opportunity to assess questions about the nature of orthographic representations in fingerspelling, as they require accurate identity, quantity, and position encoding. In written spelling, geminates are processed as distinct orthographic units and often produce characteristic errors such as omissions and mis-doublings (Caramazza & Miceli, 1990; Tainturier & Caramazza, 1996). Some examples include doubling the wrong segment (“rabitt” for “rabbit”) or deleting a geminate (“brom” for “broom”), highlighting their specialized representation (Hepner et al., 2018). Evidence from dysgraphic patients further supports the idea that geminates are not simply linear letter pairs but involve multidimensional encodings (Holmes & Carruthers, 1998; McCloskey et al., 1994).

In ASL fingerspelling, geminates are marked through distinct articulatory movements or handshape modifications, e.g., straight movement for the double “O” in P-O-O-L ^1^ or the bent “V” handshape for “ZZ” in P-I-ZZ-A (Geraci, 2009), potentially supporting more accurate preservation of geminates in fingerspelling. However, no prior study has systematically examined geminate errors in fingerspelling, making this a novel area for investigation.

In this study, we adapted the VL2 Fingerspelling Reproduction Test (Morere & Allen, 2012) to examine fingerspelling errors in deaf ASL signers. The stimuli varied in orthographic complexity and included items with geminate segments, allowing us to examine how different orthographic features influence error patterns across modalities. A comparison group of hearing non-signers completed an audiovisual dictation task with the same stimuli and typed responses. The inclusion of a hearing group was motivated by long-standing questions about how phonological knowledge influences spelling errors. Since deaf and hearing participants differ in their access to speech-based phonology, we expected group differences in error types (e.g., “the number of pronunciation-preserving vs. violating errors”) rather than overall spelling accuracy.

Although fingerspelling and typing both engage orthographic retrieval, manual articulation, and serial production, we acknowledge that these response types are not completely comparable. Our primary aim was to analyze modality-specific spelling strategies, not to equate the tasks. We wanted to use the fingerspelling reproduction test in part to allow for the possible future comparisons of fingerspelled errors with previous research that used this task (Morere & Allen, 2012). The typing task for hearing participants also approximates the sequential and motoric demands of fingerspelling while minimizing the variability introduced by handwriting fluency and legibility among writers. These methodological decisions allowed us to investigate how orthographic encoding differs across populations and modalities.

We hypothesized that:

1. Deaf signers would produce more pronunciation-violating errors, reflecting weaker reliance on phoneme-to-grapheme mapping. However, because fingerspelling is often accompanied by mouthing, some phonological influence may still emerge, for example, in visually ambiguous visemes (e.g., dropping the “a” from the “ea” segment in “appearance”).
2. Deaf signers would produce more transpositions and letter shifts compared to other error types than hearing non-signers, which would suggest weaker positional encoding. This prediction is based on the transient nature of fingerspelling and coarticulation across handshapes, which may reduce the salience of exact letter order.
3. Deaf signers would produce more deletions in fingerspelling, particularly in sequences involving complex handshape transitions. Fingerspelling requires fine motor coordination, and during rapid fingerspelling, some handshapes may be omitted (Quinto-Pozos, 2010).
4. Deaf signers would show better preservation of geminates compared to hearing non-signers, due to explicit visual-motor encoding of double letters in ASL fingerspelling.

Our stimuli included pseudowords to examine whether deaf signers apply consistent visual-motor strategies to unfamiliar letter strings without established lexical representations or whether lexical familiarity supports more accurate production. If deaf signers produce similar error rates and error types (e.g., “deletions, transpositions”) for pseudowords as for real words, this pattern would indicate consistent strategies regardless of lexical representation. For hearing participants, pseudowords were not analyzed due to variability in acceptable phoneme-to-grapheme correspondences. Finally, some of the real words on the VL2 list did not have a lexical sign equivalent in ASL. We thus explored whether fingerspelling might be more accurate if the concept is always fingerspelled because a lexical ASL translation does not exist.

## Methods

### Participants

A total of 39 deaf adults participated in the study (24 women; *M* age = 35 years, SD = 10; range = 23–61 years). All were prelingually deaf and reported profound to severe hearing loss, with an average onset of deafness at 7 months of age. One participant reported progressive hearing loss by age 10. All deaf participants identified ASL as their primary and preferred mode of communication. The average age of ASL exposure was 17 months (SD = 20 months); 22 participants acquired ASL from birth and had at least one deaf parent or caregiver. A comparison group of 35 hearing English monolinguals also participated (20 women; *M* age = 25 years, SD = 10; range = 19–62 years). All participants reported no history of neurological or learning disabilities and had normal or corrected-to-normal vision. Deaf participants were recruited via the laboratory’s participant database and contacts in the deaf community. Hearing participants were recruited through personal contacts and classroom announcements. All participants were compensated for their time.

As a baseline measure of spelling ability, all participants completed a receptive spelling test (Andrews & Hersch, 2010). The test included 88 items, half correctly spelled, and half misspelled (e.g., “*addmission, *seperate”) presented in column format on a paper sheet. Participants were instructed to identify and circle incorrectly spelled items. Scoring was based on the number of correctly classified items (i.e., hits and correct rejections). The two groups did not differ significantly in spelling recognition performance: deaf participants scored an average of 73 items correct (83%), and hearing participants scored 76 (86%), *p* = .167.

### Stimuli

The experiment included a total of 84 items: 57 real English words and 27 pseudowords, with an average length of six letters (range: 2–13 letters). Forty-five real words and 25 pseudoword stimuli were items in the VL2 Fingerspelling Reproduction Test (Allen & Morere, 2012). An additional 12 real words and 2 pseudowords were added to complete two experimental lists, balanced for lexical frequency and letter length. These supplemental items included commonly misspelled words and words with geminate segments selected based on previous studies (Hanson, 1982; Hayes et al., 2011; Olson & Caramazza, 2004). Although pseudoword spelling errors were not analyzed in the hearing group, pseudowords were included in both lists to ensure equivalence across lists and tasks.

Sixteen of the real-word items contained a geminate segment. Across all items, the average length was 6 letters (range: 2–13 letters), and the average log-transformed lexical frequency (Subtlex-US) for real words was 3.16. Items were pseudo-randomly assigned to two lists of 42 items each, to allow participants a break midway through the session. Lists were matched for word length and frequency and included a similar number of pseudowords. Thirty-two of the words had a lexical equivalent in ASL (e.g., “cough”) and 25 did not (e.g., “soliloquy”), and thus, these concepts would typically be fingerspelled. A female deaf signer, who acquired ASL from birth from deaf parents, determined whether an item would be typically fingerspelled.

The same deaf signer modeled fingerspelled items at her natural signing pace with her dominant right hand. Video recordings captured the model’s face, arms, and torso in frame, and were edited into individual video clips using Final Cut Pro^™^. Each fingerspelled clip was approximately three seconds in duration. Although fingerspelling often co-occurs with mouthing, the signer was encouraged to keep mouthing to a minimum during fingerspelling productions so that the stimuli paralleled the productions in the VL2 fingerspelling test (Morere & Allen, 2012). By excluding overt mouthing, we eliminated variability across signers with respect to the use of mouthing as a spelling cue, given that mouthing during fingerspelling is highly variable across signers (Nadolske & Rosenstock, 2007). For the hearing participants, an audiovisual version of the same stimuli was created. Each item was spoken by a female monolingual English speaker using a North American accent, recorded with her face and torso visible. Audiovisual clips were edited to match the fingerspelled clips in duration and presented in the same item order across both modalities. The stimulus lists and videos are shared in the Supplementary Materials on Open Science Framework (OSF): https://doi.org/10.17605/OSF.IO/R3G2P.

### Procedure

The experiment was completed individually in a quiet room with minimal visual and auditory distractions. Stimuli were presented using Qualtrics^™^ on a Mac desktop computer in a self-paced format. Deaf participants received instructions in both written English and ASL, while hearing participants received instructions in written and spoken English. Participants were instructed to wait until the video finished playing before producing their responses. Deaf participants responded by repeating the fingerspelled word to a video camera at their natural fingerspelling rate, while hearing participants wore headphones and typed the word they heard and saw at their natural typing speed.

Participants advanced through the experiment at their own pace by pressing a key after each response. Hearing participants could press backspace to immediately correct their typing, but once they submitted the response they could not go back. This procedure was the same for deaf participants who could have corrected their response, e.g., false starts for both typing and fingerspelling were allowed, but excluded from the analysis. Each participant completed 85 trials (including one practice item), presented in two blocks to allow for a half-way break. Stimuli were presented in a fixed pseudo-randomized order, and each item was shown once.

### Data coding and analysis

Video recordings of fingerspelled responses were transcribed by a deaf early ASL signer and entered into a CSV file. Hearing participants’ written responses were exported as a CSV file from Qualtrics^™^. Alternative British English spellings (“mustache” vs. “moustache” and “neighbor” vs. “neighbour”) were accepted as correct responses. Trials where the participant missed the prompt and did not provide a response were excluded from analyses. We retained trials where a clear attempt was made to spell the correct target word, and at least 40% of the target word spelling was preserved in the response (these were coded as incorrectly spelled trials). Such responses included the following: “damite” for “diamond,” “brochi” for “broccoli,” “conious” for “conscious,” “nebjor” for “neighbor,” or “aspohe” for “apostrophe.” False starts and repeated responses were excluded. We calculated average accuracy for correctly spelled trials (% accuracy). All remaining, incorrectly spelled trials were further analyzed for error types.

#### String edit distance

We next estimated the string edit distance using the Damerau–Levenshtein Distance (DLD) calculations for each target-response pair which measures the number of operations needed to correct spelling errors. The DLD weights string edit distance for production errors such as substitution, deletion, insertion, as well as transposition errors (see also Yarkoni et al., 2008) and provides a nuanced analysis of spelling patterns. Larger DLD values indicate that the participants’ internal orthographic representations differ greatly from the target spellings. The DLD values positively correlated with the manual error counts (*R*^2^ = .895, *p* < .001) ensuring metric reliability.

#### Error type scoring

Three researchers independently coded all incorrect responses according to pre-defined spelling error categories (see Table 1). Error classification was based on the taxonomy developed by Olson and Caramazza (2004) which allows us to distinguish between position-preserving errors (substitutions, omissions, insertions) and position-violating errors (letter shifts, adjacent transpositions and non-adjacent transpositions). In addition to these structural error types, we coded pronunciation-violating errors, defined as responses that resulted in either unpronounceable letter strings or misspellings that deviated from the American English pronunciation of the target word (e.g., “subsuite” for “substitute,” “reptitles” for “reptiles,” or “couhg” for “cough”). Morphological insertions that preserved pronunciation, such as adding or doubling a plural marker (e.g., “scissorss” or “broccolis”), were not classified as pronunciation-violating errors but were coded as insertions. In contrast, deletions of essential plural markers on mass nouns (e.g., “scissor” for “scissors”) were coded as both a pronunciation-violating error and a deletion.

We also coded for errors involving geminate segments (e.g., “bb,” “oo”), focusing on whether the double-letter segments were preserved, fully or partially. Both insertions (e.g., “rabbitt” for “rabbit”) and deletions (e.g., “brom” for “broom”) were categorized accordingly. Multiple errors or error types could be assigned per response, for example, “soliloquily” for “soliloquy” was coded as two insertions (“i” and “l”), while “congail” for “congenial” was scored as two deletions (“e” and “n”) and one adjacent transposition (“ai” for “ia”). In such cases, the total error count also included a pronunciation-violating error if the misspelling altered the phonological integrity of the target word.

To establish inter-rater reliability, two independent researchers double-blind coded 45% of the incorrectly spelled responses. Agreement between the two coders was 93.7%. A third researcher (one of the authors) reviewed all discrepancies, and the remaining error coding was resolved through discussion. Representative examples of each error type for both fingerspelled and written responses from our data are provided in Table 1. All errors and error counts are provided in a table in Supplementary Materials on OSF.

## Results

The results include analyses of real words only (Tables 2-4). Deaf participants were accurate on 87% of real-word fingerspelling trials while hearing participants were accurate on 88% of real-word spelling trials. A binary logistic regression revealed no significant group difference in spelling accuracy, *B* = .085, *SE* = .095, *p* = .370, log-likelihood = −1538.

To assess letter-level accuracy, we analyzed string edit distance (DLD). A linear mixed-effects regression using the lmer package in R (Bates et al., 2015) showed no significant difference in DLD between groups. Deaf participants had an average DLD of .302 (*SD* = .952), while hearing participants had an average DLD of .231 (*SD* = .714), *B* = −.075, *SE* = .045, *t* = −1.683, *p* = .097. This result represents a small-to-moderate effect size, Cohen’s *d* = .351.

Error count (sum of substitution, insertion, deletion, and position-violating errors) was also comparable across groups. Deaf participants produced a total of 996 errors, and hearing participants produced 874 errors. A linear mixed-effects regression confirmed no significant group effect, *B* = −.032, *SE* = .075, *t* = –.430, *p* = .669.

To examine the relationship between spelling accuracy and spelling knowledge, we correlated each participant’s DLD score with their performance on the receptive spelling test (Andrews & Hersch, 2010). No significant correlations were found for either group: deaf participants, *r* = −.245, *p* = .134; hearing participants, *r* = .29, *p* = .086. To sum up, deaf and hearing participants performed with comparable overall accuracy on their respective spelling tasks, fingerspelling reproduction, and written dictation, with no significant group differences in accuracy, string edit distance, or total error count.

Next, we examined the distribution of spelling error types (substitution, insertion, deletion, and position-violating errors) across groups. An analysis of variance on error count revealed no main effect of group, *F* (1, 295) = 1.59, *p* = .209. There was a significant main effect of error type, *F* (3, 295) = 10.70, *p* < .001, and a significant Group × Error Type interaction, *F* (3, 295) = 14.20, *p* < .001. We followed up with linear mixed-effects models comparing deaf and hearing groups on each error type, using participant and item as random intercepts (Table 2).

Figure 1 illustrates that deaf and hearing participants differed significantly on all error types except insertions. Deaf participants produced more deletions and position-violating errors, while hearing participants made more substitution errors. For the subtypes of position-violating errors, deaf participants produced 80 letter shifts, 46 adjacent transpositions, and 27 non-adjacent transpositions. In contrast, hearing participants produced only 8 letter shifts, 12 adjacent transpositions, and 4 non-adjacent transpositions. Due to low frequencies, statistical comparisons for these subtypes were not performed.

Regarding geminate errors, deaf participants made significantly fewer errors than hearing participants. Deaf participants preserved double-letter sequences more accurately with respect to both geminate deletions (*M* = .015, *SD* = .017) and insertions (*M* = .011, *SD* = .020), compared to hearing participants (*M* deletions = .032, *SD* = .021; *M* insertions = .030, *SD* = .024). Linear mixed-effects analyses revealed significant group differences for both deletion errors, *B* = .017, *SE* = .004, *t* = 3.77, *p* = .0003, *d* = .871, and insertion errors, *B* = .020, *SE* = .005, *t* = 3.78, *p* = .0003, *d* = .877, both large effect sizes.

Finally, deaf participants made significantly more pronunciation-violating errors than hearing participants. These included substitutions, deletions, and transpositions that altered the phonological form of the target word. Effect sizes for all significant comparisons ranged from moderate to large (see Table 2, Cohen’s *d*). Table 3 presents the top ten most frequently misspelled words in each group, and Table 4 provides examples of productions that were unique to each group (i.e., only occurred in that group or disproportionately occurred in that group).

To examine whether lexical status influenced fingerspelling performance, we compared deaf participants’ accuracy on real words versus pseudowords (see Table 3). Accuracy was equivalent across stimulus type: deaf participants correctly reproduced both real words (*M* = 87%, *SD* = 34%) and pseudowords (*M* = 87%, *SD* = 34%), with no significant difference, *p* = .810. String edit distance (DLD) was also comparable across words and pseudowords. Average DLD for pseudowords was .27 (*SD* = .82) and for real words was .30 (*SD* = .95). A linear mixed-effects regression predicting DLD from lexical status (real vs. pseudoword) revealed no significant effect, *B* = .026, *SE* = .038, *t* = .684, *p* = .496. Error rates by type were similar between real and pseudoword items (Table 3). None of the differences in substitution, deletion, insertion, position-violating, geminate, or pronunciation-violating errors reached statistical significance (all *ps* ≥ .118).

Finally, we examined whether the existence of a lexical ASL translation equivalent to the English word influenced fingerspelling accuracy. Deaf participants fingerspelled words with an ASL translation more accurately (*M* = 91%, *SD* = 29%) than words without an ASL translation (*M* = 83%, *SD* = 38%), but this difference was not statistically significant (*p* = .05). A comparison of string edit distance (DLD) revealed a similar pattern: Words with ASL translations had lower DLDs (*M* = .24, *SD* = .90) than those without (*M* = .39, *SD* = 1.00). However, a linear mixed-effects regression with DLD as the outcome and ASL translation equivalence as the predictor confirmed that this difference was not significant, *B* = −.14, *SE* = .17, *t* = .84, *p* = .403. Regression analyses on individual error types also showed no significant differences between the two word types across all error categories (see Table 4). Words with an ASL translation equivalent and words that are typically fingerspelled were produced with comparable accuracy and similar error patterns.

## Discussion

Deaf and hearing participants demonstrated similar accuracy in their respective spelling tasks, with no significant differences in overall error count or string edit distance. However, the types of spelling errors differed notably between groups. Deaf signers produced significantly more deletions and position-violating errors when fingerspelling, while hearing speakers made more substitution errors when writing to dictation. As expected, deaf participants made significantly more pronunciation-violating errors than hearing participants, replicating previous studies of written spelling (Gärdenfors et al., 2019; Hanson et al., 1983; Olson & Caramazza, 2004). Of particular interest, deaf participants outperformed hearing participants on the production of geminates (double letters), producing fewer geminate deletions and insertions. This pattern may arise due to the marked articulation of double letters in fingerspelling, a salient feature that could support geminate preservation in fingerspelling. An analysis of pseudowords (vs. real words) and items with and without ASL translation equivalents revealed that neither lexical status nor the existence of sign translations significantly affected fingerspelling accuracy or error patterns. Deaf signers seemed to apply similar spelling strategies for familiar items (real words and words that are typically fingerspelled) and unfamiliar items (pseudowords and words that have sign translations and thus are not typically fingerspelled) with no measurable decline in accuracy or shift in error types. Thus, fingerspelling reproduction relies on general orthographic and visual-motor encoding mechanisms, rather than stored word forms alone.

The first key finding, a similarity in overall accuracy and string edit distance between groups, aligns with prior research showing that deaf signers, particularly those with early exposure to ASL, demonstrate spelling accuracy comparable to hearing peers (Daigle et al., 2020; Gärdenfors et al., 2019; Miller et al., 2021; Vizzi et al., 2022). These studies have challenged the assumption that reduced access to phonology necessarily leads to poorer spelling outcomes in deaf populations. Instead, our findings support the view that alternative strategies, especially visual-orthographic encoding mechanisms, can support spelling performance in the absence of robust speech-based phonological representations.

Secondly, we found that the qualitative nature of the errors differed between the groups. Deaf participants exhibited higher rates of transpositions and deletions whereas hearing participants produced more substitutions. This group difference could reflect less flexibility in serial position for the hearing participants compared to deaf participants. That is, hearing participants maintained the letter position but substituted a similar sounding or a visually similar letter, often without a change in the target pronunciation. For example, substituting the “e” in “apostrophe” for “y” as in “apostrophy,” or substituting “o” in “apostrophy” with an “a” as in “apostraphy.” It is important to note that these error types (transpositions, deletions, and substitutions) are precisely those that Krajenbrink et al. (2021) associate with increased working memory load and graphemic buffer difficulties in hearing speakers, especially in longer or more complex words. The graphemic buffer, a component of working memory, holds orthographic representations active while the act of spelling takes place and plays a key role in spelling production (Caramazza & Miceli, 1990; Krajenbrink et al., 2021). We conducted a post-hoc correlation between the word character length and error rates to investigate whether this pattern holds for fingerspelling, as well as for written spelling. The analysis for fingerspelling revealed a positive moderate correlation between length and deletion error rates (*r* = .342, *p* < .001; 95% CI [.304; .379]) as well as position-violating error rates (*r* = .288, *p* < .001, 95% CI [.249; .327] (correlation between length and all error rates was *r* = .394, *p* < .001). For hearing participants, the correlation between length and deletion rates (*r* = .244, *p* < .001, 95% CI [.203; .284]) and position-violating error rates (*r* = .115, *p* < .001; CI [.073; .157]) was also present but somewhat weaker (correlation between length and all error rates was *r* = .260, *p* < .001; CI [.219; .299]). We suggest that longer, more complex sequences, especially in fingerspelling, impose greater processing demands on working memory and that fingerspelling representations may be held in a manual graphemic buffer.

Research on the graphemic buffer shows that phonological similarity between characters can disrupt the serial maintenance of letter sequences during spelling (Tainturier & Rapp, 2010). Extending this idea to the manual modality, we propose that sign-based phonological similarity, specifically, manual articulatory similarity between fingerspelled handshapes, may similarly destabilize letter representations within the graphemic buffer during fingerspelling production. In our dataset, errors such as the deletion of the second “O” in B-R-O-C-C-O-L-I (where the fingerspelled letters “O” and “C” share visual/manual similarity) or dropping the second “A” in A-P-P-E-A-R-A-N-C-E (where “A” and “E” share visual/manual similarity) involve a transition between two visually/manually similar handshapes (Richards & Hanson, 1985). Such errors may reflect similarity-based interference in the graphemic buffer, making similar segments more vulnerable to deletions or transpositions. In addition to buffer-level similarity effects, coarticulation or motor assimilation during rapid fingerspelling likely contributes to deletion or transposition error patterns. Rapid fingerspelling can lead to articulation favoring transitions between similar handshapes (Quinto-Pozos, 2010). The deletion of “R” between two compact handshapes “T” and “O” in A-P-O-S-T-O-P-H-E could make the non-compact handshape “R” more vulnerable to deletion due to markedness differences. Deletions and transpositions in fingerspelling may arise from similarity-based interference in planning or reflect motor adaptations during articulation (readout), as signers prioritize efficient handshape transitions over preserving precise forms.

Whether two handshapes are considered similar depends largely on the model of handshape similarity being applied. Perceptually driven models, such as that of Richards and Hanson (1985), rely on signer judgments about visual or production-based similarity. In their study, nearly half of participants grouped “T” and “O” together, suggesting that these handshapes may appear similar in overall shape or compactness. In contrast, articulatorily grounded models of phonological similarity derive similarity from joint angles and phonological feature overlap (Keane et al., 2017). Under Keane’s model, “T” and “O” are not treated as highly similar due to their differences in aperture and selected fingers. This divergence underscores that handshape similarity is model-dependent and remains an open empirical question. It also highlights the importance of using theory-driven metrics to systematically evaluate similarity in fingerspelling tasks.

Flexible, less strict letter position ordering may also be more tolerated in dynamic fingerspelling than print because the speller/perceiver can mentally “autocorrect” a letter swap, which is not possible for static written representations. Under time constraints, this flexibility may lead to rapid “decay” of orthographic memory for fingerspelled words and an increase in positional instability (Fischer-Baum et al., 2010). Fingerspelling speed has been shown to affect articulation precision: faster fingerspelling increases the likelihood of transpositions due to the difficulty of maintaining accurate sequential encoding under time pressure (Quinto-Pozos, 2010).

Further, fingerspelling is not merely the articulation of individual static handshapes; it is a process of transitions among handshapes that create a fluent sequence or contour (Padden & Le Master, 1985). Under this perspective, the higher deletion and transposition error rates in fingerspelling than print may reflect pressures to optimize transitional movements during fluent production. Maintaining efficient transitions between visually and manually similar handshapes could lead to the suppression or skipping of more complex, distinct handshapes or cluster reduction that would otherwise disrupt motoric fluency. This framework supports the idea that articulatory dynamics, and not just memory load, shape the ultimate form of output during fingerspelling repetitions.

A third important result was that deaf participants produced significantly more pronunciation-violating errors in fingerspelled productions than hearing participants in written productions, further supporting the view that phonological information plays a reduced role in deaf signers’ orthographic representations. The fingerspelled errors often resulted in nonwords or orthographic forms that deviated from standard English pronunciation (e.g., “unaminous” for “unanimous,” “manison” for “mansion,” “viligant” for “vigilant”). These errors indicate that fingerspelling is less constrained by sound-to-letter mappings compared to the written output of hearing participants. Several previous studies have found a similar error pattern in written productions by deaf participants. As noted in the introduction, Olson and Caramazza (2004) reported that most written spelling errors by deaf participants violated pronunciation compared to hearing participants. The fact that many pronunciation violations are also found in fingerspelling suggests that deaf signers rely more on visual-orthographic and motoric encoding strategies, which do not necessarily preserve the phonological structure of words. For example, a common fingerspelling error was producing U-N-A-M-I-N-O-U-S for U-N-A-N-I-M-O-U-S. In this case, the visually/manually similar letters “M” and “N” are transposed (for reasons discussed above). This non-adjacent transposition violates pronunciation but maintains visual-orthographic plausibility.

Another compelling pronunciation-violating example was a common fingerspelling error M-A-N-I-S-O-N for M-A-N-S-I-O-N, in which the target word’s complex consonant cluster (CCV) is reshaped into a more frequent and perceptually “regular” CVCV syllable pattern, while letter identity is preserved within the word. In this case, moving the handshape representing the vowel “I” between the consonants “N” and “S” indicates a preference for alternating CV structures, which are common in English and visually easier to segment in fingerspelling. Deaf signers may therefore regularize unfamiliar or complex orthographic structures by aligning them with more familiar orthographic templates, even if the result violates pronunciation. These transposition errors underscore how deaf signers may restructure unfamiliar words not based on how they sound, but on how they *look* or *feel* in fingerspelling production. This type of visual-orthographic regularization further highlights how reduced phonological access leads to distinct, visually driven spelling strategies.

Finally, a fourth key result was the deaf participants’ superior performance in preserving geminate (double letter) segments compared to the written productions of hearing participants. This finding provides compelling evidence that fingerspelling supports robust visual-orthographic encoding of both letter identity and quantity. We observed that deaf participants made significantly fewer errors on geminate sequences than hearing participants, both in terms of deletions and insertions (see Table 3 for examples). This pattern suggests that fingerspelling provides stronger support for encoding geminate structure than phonologically mediated spelling. Whereas hearing speakers rely on phonological forms that do not typically distinguish between single and double consonants or vowels, deaf signers access a visual–manual representation in which geminates are explicitly marked through repeated movements. For example, in the fingerspelled word S-Y-L-L-A-B-L-E, the repeated “L” is conveyed in ASL fingerspelling with a straight movement between the repeated handshapes, creating a salient visual and motoric cue that reinforces the identity and quantity of the double letter. This unique representational feature of fingerspelling may strengthen the mental encoding of geminates as distinct from single-letter counterparts.

This finding supports theoretical models of orthographic representation that treat letter identity, quantity, and position as independently encoded dimensions (Caramazza & Miceli, 1990; Fischer-Baum & Rapp, 2014). Hearing participants were significantly more likely to omit or erroneously insert geminates, which aligns with previous work showing that phonological mediation provides weak support for distinguishing geminate segments (Hepner et al., 2018). Geminate preservation by deaf participants is also consistent with Gärdenfors et al. (2019) who observed that in written spelling, deaf signers of Swedish Sign Language produced fewer geminate errors than hearing non-signers. We suggest that there are two possible reasons that deaf signers preserve geminates in written and fingerspelled outputs: a lack of speech-based phonological influence and the visual and motoric marking of geminate segments required by fingerspelling. These factors allow deaf signers to more consistently preserve geminate structure. This result highlights how visual–manual encoding may benefit the representation of complex orthographic structures.

Additionally, we analyzed fingerspelling reproduction of pseudowords vs. real words. The finding that deaf signers reproduced pseudowords with similar accuracy and error patterns as real words suggests that fingerspelling utilizes general orthographic encoding strategies and does not rely solely on stored word-specific representations. This finding aligned well with the result that fingerspelling accuracy and error types were not different for items that are typically signed (have an ASL sign equivalent) versus items that are typically fingerspelled (do not have an ASL sign). Therefore, fingerspelling accuracy does not appear to be contingent on prior lexical knowledge of the item. The ability to just as accurately reproduce unfamiliar pseudowords implies that deaf signers’ orthographic representations and production skills are rule-based and flexible, drawing on consistent visual-motor encoding mechanisms.

Unfortunately, few empirical studies have systematically analyzed fingerspelling error patterns, and most focus on ASL, making comparisons across studies or generalizations about error patterns to other sign languages problematic. While some have described patterns of articulatory reduction or coarticulation in rapid fingerspelled productions (Keane et al., 2012; Quinto-Pozos, 2010; Wilcox, 1992) and production or perception-based similarity handshape metrics have been proposed (Keane et al., 2017; Richards & Hanson, 1985), fingerspelling error rates or types have been scarcely studied. This highlights an urgent need for cross-linguistic research and standardized frameworks for comparing fingerspelling errors across sign languages.

### Limitations and future directions

One limitation of this study is that fingerspelling and typing are not completely parallel, and it is possible that some errors may have been influenced by the response mode. For example, some typing errors could have been influenced by the keyboard layout, and some fingerspelling errors could have been influenced by articulation demands (e.g., “handshape complexity”). One way to partially overcome this limitation is to equate the response mode across groups by asking deaf signers to write or type the fingerspelled word, rather than to repeat it. Nonetheless, the error patterns that we observed for fingerspelling align with previous reports of deaf written spelling (Aaron et al., 1998; Gärdenfors et al., 2019; Olson & Caramazza, 2004), which document frequent deletions and transpositions, especially in complex words. Parallels between fingerspelling and written spelling errors suggest that position coding is a flexible aspect of orthography for deaf signers across both modalities, but this idea warrants further research.

## Conclusion

This study revealed that deaf signers achieve spelling accuracy comparable to hearing speakers but differ qualitatively in error patterns. Deaf participants produced more deletions and transpositions, linked to graphemic buffer demands and motoric fluency pressures, whereas hearing participants made more substitutions, reflecting a greater propensity to preserve position. Errors involving manual similarity and articulation dynamics suggest that fingerspelling places unique constraints on orthographic encoding. Notably, deaf participants preserved geminate structures more successfully than hearing participants, highlighting the visual–manual advantages of fingerspelling. These results underscore that deaf signers rely on flexible, visually driven strategies rather than phonological mediation, with implications for models of spelling and literacy instruction. Fingerspelling plays a central role in orthographic development and maintenance for deaf children (Lee & Secora, 2022). Future research could examine how visual, motoric, and lexical experiences interact to shape spelling outcomes in deaf signers.

## Figures and Tables

**Figure 1.**
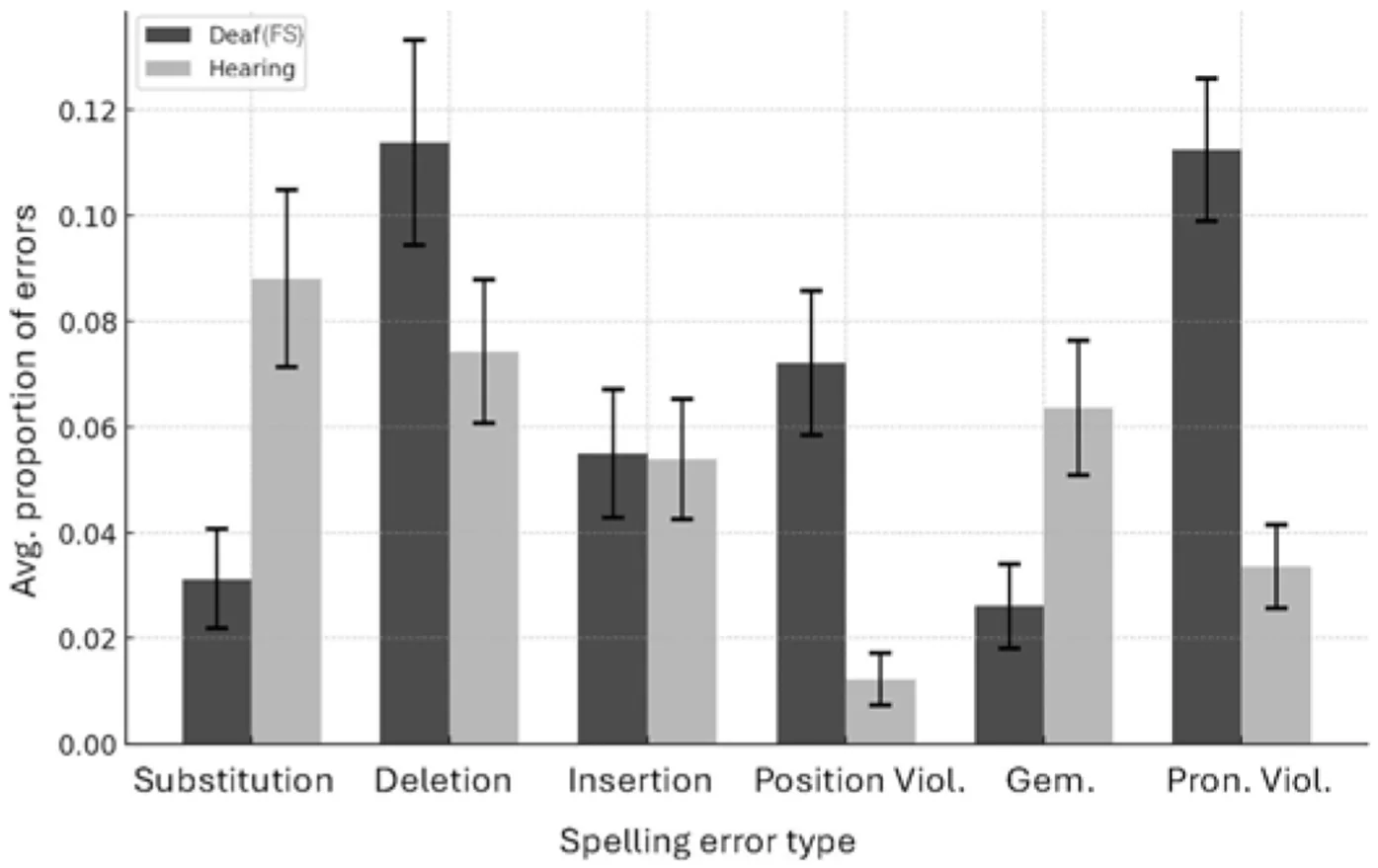
The proportion of errors for real words (*Y*-axis) with 95% confidence intervals shown for each spelling error type (*X*-axis) for deaf participants producing fingerspelling (dark gray bars) and hearing participants producing written English (light gray bars). The average proportion of error was calculated across all items. “Position Viol.” = position-violating errors (transpositions, shifts), “Gem” = geminate errors; “Pron. Viol” = pronunciation-violating errors.

**Table 1.** Error types and examples of errors by deaf signers in the fingerspelling task and by hearing speakers in the audiovisual dictation task

Category	Example from fingerspelling	Example from dictation
Position preserving errors:		
Substitution	concrete → *contrete	apostrophe → *apostrophy
Deletion	disappearance → *disapperance	omniscient → *omnicient
Insertion	treasure → *tresasure	vacillate → *vascillate
Position-violating errors:		
Letter shifts	congenial → *congienal	acquaintance → *acquinatance
Transpositions non-adjacent:	unanimous → *unaminous	congenial → *congineal
Transpositions adjacent:	mansion → *manison	acquaintance → *acquiantance
Phonological errors:		
Target pronunciation preserved	broccoli → *broccli	vacillate → *vasilate
Target pronunciation violated	congenial → *congental substitute → *subsuite	apostrophe → *aposterphy reptiles → *reptitles
Geminate errors:		
Deletion	bizarre → *bizare broccoli → *broli	inflammation → *inflamation
Insertion	omniscient → *ommniscient	soliloquy → *solliloquy

**Table 2.** Average proportion of spelling errors (M), error counts (*n*), standard deviation (SD), and linear mixed-effects regression models comparing each error type produced by deaf signers (fingerspelling) and hearing monolingual English speakers (written English)

Error type	Deaf		Hearing		Test statistics			
	M (*n*)	SD	M (*n*)	SD	Est. B (SE)	*t*	*p*	Cohen’s *d*
Substitution	.031 (66)	.222	.088 (173)	.379	.056 (.014)	3.99	.000	.945
Deletion	.114 (240)	.456	.074 (146)	.305	−.041 (.018)	−2.21	.030	−.452
Insertion	.055 (116)	.284	.054 (106)	.256	−.002 (.010)	−.18	.856	−.014
Position viol.	.072 (152)	.319	.012 (24)	.110	−.061 (.010)	−5.94	.000	1.369
Geminate	.026 (55)	.189	.064 (125)	.288	.038 (.009)	4.41	.000	1.012
Pron. viol.	.112 (237)	.316	.034 (66)	.180	−.080 (.013)	−.6.38	.000	−1.466

**Table 3.** A comparison of fingerspelling errors for real words vs. pseudowords and linear mixed-effects regression results

Error type	Real word		Pseudoword		Test statistics			
	M	SD	M	SD	Est. B (SE)	*t*	*p*	Cohen’s *d*
Substitution	.031	.222	.064	.304	−.034 (.021)	−1.579	.118	.251
Deletion	.114	.456	.087	.391	.025 (.057)	.447	.656	.437
Insertion	.055	.284	.052	.268	.004 (.026)	.140	.889	.279
Position viol.	.072	.319	.047	.230	.026 (.038)	.684	.496	.294
Geminate	.026	.189	.046	.272	−.022 (.031)	−.729	.468	.219
Pron. viol.	.112	.316	.121	.326	−.010 (.059)	−.202	.840	.320

**Table 4.** A comparison of fingerspelling errors for words with vs. without ASL sign equivalent and linear mixed-effects regression results

Error type	Sign equivalent		No sign equivalent		Test statistics		
	M	SD	M	SD	Est. B (SE)	*t*	*p*
Substitution	.024	.206	.040	.241	.016 (.019)	−.809	.422
Deletion	.112	.491	.116	.407	.002 (.070)	−.031	.975
Insertion	.035	.218	.080	.349	.044 (.030)	−1.456	.151
Violates pos.	.052	.294	.098	.347	.044 (.048)	−.919	.362
Geminate	.026	.184	.026	.196	.000 (.000)	−.007	.995
Violates pron.	.083	.277	.151	.358	.067 (.055)	−1.208	.232

## Footnotes

1 Fingerspelled words are represented in all capital letters with hyphens between each letter (e.g., F-U-N). Standard orthographic forms are presented in lower case. This typographical distinction clarifies whether we are discussing the manual-visual representation or the written linguistic form.
